# Group-Living Herbivores Weigh Up Food Availability and Dominance Status when Making Patch-Joining Decisions

**DOI:** 10.1371/journal.pone.0109011

**Published:** 2014-10-01

**Authors:** Keenan Stears, Graham I. H. Kerley, Adrian M. Shrader

**Affiliations:** 1 School of Life Sciences, University of KwaZulu-Natal, Pietermaritzburg, South Africa; 2 Centre for African Conservation Ecology, Department of Zoology, Nelson Mandela Metropolitan University, Port Elizabeth, South Africa; Queen Mary, University of London, United Kingdom

## Abstract

Two key factors that influence the foraging behaviour of group-living herbivores are food availability and individual dominance status. Yet, how the combination of these factors influences the patch-joining decisions of individuals foraging within groups has scarcely been explored. To address this, we focused on the patch-joining decisions of group-living domestic goats (*Capra hircus*). When individuals were tested against the top four ranked goats of the herd, we found that at patches with low food availability they avoided these dominant patch-holders and only joined subordinates (i.e. costs outweighed benefits). However, as the amount of food increased, the avoidance of the top ranked individuals declined. Specifically, goats shifted and joined the patch of an individual one dominance rank higher than the previous dominant patch holder when the initial quantity of food in the new patch was twice that of the lower ranking individual’s patch (i.e. benefits outweighed costs). In contrast, when individuals chose between patches held by dominant goats, other than the top four ranked goats, and subordinate individuals, we found that they equally joined the dominant and subordinate patch-holders. This joining was irrespective of the dominance gap, absolute rank of the dominant patch-holder, sex or food availability (i.e. benefits outweighed costs). Ultimately, our results highlight that herbivores weigh up the costs and benefits of both food availability and patch-holder dominance status when making patch-joining decisions. Furthermore, as the initial quantity of food increases, food availability becomes more important than dominance with regard to influencing patch-joining decisions.

## Introduction

Living in groups has two broad benefits: reduced predation risk and increased foraging efficiency [1], [2]. Reduction in predation risk comes from increased group vigilance [3], smaller domains of danger [4] and the dilution effect [5]. Alternatively, foraging benefits are obtained via social information [6], [7]. For example, by watching conspecifics, individuals can increase their ability to find and assess the quality of food patches [8], [9]. However, this may lead to individuals joining patch-holders at their patches, resulting in costs such as increased competition and greater social conflicts between group members [10]–[12].

One factor that can influence patch-joining decisions is the dominance status of the patch-holder [13], [14]. Dominant individuals tend to be larger and more aggressive than subordinates and thus can better defend patches [13]. However, social bonds can also influence foraging behaviour because related individuals can form subgroups [15], which can influence food preference [16] and the foraging location of group members [17], [18], with forgers spending more time feeding closer to other herd members. In addition, patch quality also influences joining decisions, as foragers prefer to feed from high quality patches [19]. Furthermore, patch quality determines the amount of food that is available to joining individuals once the patch-holder has removed the finder’s share (i.e. proportion of food available to the patch-holder before another individual arrives) [20]. In poor quality patches, less food is available to all individuals feeding within a patch, compared to high quality patches. Therefore, to obtain food in poor quality patches, patch-holders tend to defend these patches more aggressively [21].

One of the most important requirements for foragers is to feed in a manner that promotes efficiency and reduces perceived risk. Generally, the value of a patch is enhanced as the abundance of food increases [22] and decreases as the risk of foraging in that patch increases [23]. For example, Shrader *et al*. [24] found that goats reduced their feeding effort from patches that were close to predator cues (dung and urine) compared with patches that did not have predator cues (i.e. high-risk and low-risk patches). Interestingly, gerbils (*Gerbillus allenbyi* and *G. pyramidum*) increased their use of risky patches when the amount of food within these patches was about 4–8 times richer than safer patches [25]. Thus, a forager should feed in patches where it makes a trade-off between high food reward and safety [26].

In group-living animals, dominant individuals tend to aggressively defend resources, so joining a dominant individual will have higher risks compared to joining a subordinate. However, competition may also be a passive process, operating through the avoidance of conflict by subordinates [27]. Various studies have shown that individuals only join patches held by subordinate individuals [12], [14]. However, in some instances, dominance can have little effect on tactic use [28], [29]. This may be a result of the flexibility and consistency in tactic use across taxa [30]. For example, a study on Japanese macaques (*Macaca fuscata fuscata*) found that when food is abundant, patch choices by subordinates are unaffected by whether the patch is occupied by a dominant or subordinate individual [31]. This suggests that the role of dominance in foraging decisions may be influenced by additional factors such as patch quality [32]. Interestingly, the factors that influence joining decisions in species where dominance is not important are poorly known [30].

Surprisingly, the degree to which herd members weigh up food availability in a patch, and the dominance status of a patch-holder, when making patch-joining decisions, has scarcely been explored in species other than birds [12]. Goats provide a good species with which to test this as they have a linear hierarchy [33], [34], use social [9] and personal information [35], and dominant goats join lower ranking individuals at patches [33], [36]. As a result, we conducted two experiments to focus on how these two factors influence the patch-joining decisions of a group-living herbivore–the domestic goat (*Capra hircus*). The first experiment focused on how the dominance rank of the top ranked goats in the herd and food availability (i.e. the quantity of food in a patch) influenced patch-joining decisions. To further explore the influence of dominance and food availability, we ran an additional experiment that tested three hypotheses: 1) the dominance gap between a dominant patch-holder and a joining goat is important in determining patch choice, 2) the absolute rank of the dominant patch-holder will influence patch choice, and 3) the sex of the dominant patch-holder as well as the sex of the joining goat will influence patch choice. For the above experiments, if dominance is the driving force behind patch choice, we expect goats to join subordinate patch-holders, irrespective of the amount of food. Alternatively, if food availability is the main factor influencing patch choice, goats should feed from patches with large quantities of food, irrespective of the dominance status of the patch-holder.

## Materials and Methods

### Ethical statement

We adhered to the protocols of the University of KwaZulu-Natal’s Animal Ethics Committee, which cleared all animal husbandry and experimental procedures (permit number: 068/10/Animal). Prior to approval, the application was reviewed by the South African National Council of the Society for the Prevention of Cruelty to Animals (NSPCA). Throughout all the experiments, every effort was made to minimise the harm and stress to the goats, and no goats were injured during the experiments. All data are available in data supplement S1.

### Experimental procedure

Experiments ran from April 2010 to May 2011 at the Ukulinga Research Farm, Pietermaritzburg, South Africa. We used a single herd of 45 indigenous veld goats (16♂, 29♀, average age: 26±12 months), thus all the goats were familiar with each other prior to the start of our experiments. For our experiments, we relied on the goats’ use of personal information [35], [37] and their ability to remember complicated foraging tasks [35] to differentiate and remember the amount of food found in the different patch types (see below).

For eight months prior to running the experiments, the goats were exposed to the testing arenas and all the food patches to allow them to become accustomed to the experimental protocol. This habituated the goats to the different food patches and allowed them to determine the amount of food in each patch (see below). During this time, we also tested the dominance hierarchy and determined whether goats could differentiate between the different amounts of food in each patch.

Goats were housed in a 30×12 m sheltered barn containing water troughs. To ensure that the goats joined patches and fed during the experiments, we did not provide the goats with food overnight. However, they had *ad libitum* access to water. This mimicked common husbandry practices [24]. Each day prior to running the experiments, the goats were allowed to feed for an hour in a 30×15 m rye grass pasture. This limited the possibility that hunger levels would differ substantially between the first and last goats tested each morning. Experiments ran from 7 h00 to 11 h00 each day. After running the experiments, all the goats were released to forage in a natural grassland for the remainder of the day (i.e. ∼6 hours).

### Dominance relationships

Prior to all experiments, we determined the dominance hierarchy of the herd (Table 1). Initially, we explored the possibility of recording natural agonistic interactions between herd members as a way to estimate the hierarchy because this would limit any added stress or injury to the animals. However, after initial observations, we concluded that it would take an inordinate amount of time to observe interactions between all the different individuals (*N* = 45 goats). This is because each day the goats fed in a large natural grassland where our ability to observe them was limited. Thus, due to the impracticalities of observing interactions in the field, we chose to stage the interactions experimentally.

**Table 1:** Sex, presence of horns, and dominance rank of the goats calculated using David’s Score.

Goat name	Sex	Horned	Dominance rank	David’s score
Blue 05	M	Yes	1	977
IG35	M	Yes	2	932
Blue06	M	Yes	3	929
IG41	M	Yes	4	842
Big beige	M	Yes	5	797
Blue04	M	No	6	720
IG1	F	Yes	7	708
Matey	F	Yes	8	619
Mottled	F	Yes	9	618
IG36	M	Yes	10	485
IG32	F	No	11	484
Blue03	F	Yes	12	444
IG22	F	No	13	443
IG42	M	No	14	348
IG46	M	Yes	15	336
Blue01	F	No	16	309
IG51	M	No	17	260
Blue08	M	No	18	233
IG57	M	Yes	19	220
S6K	F	No	20	174
S3K	F	No	21	140
Blue02	F	Yes	22	−4
S2K	F	No	23	−5
S7K	F	No	24	−50
TK1	M	Yes	25	−77
TK2	M	Yes	26	−122
Blue07	F	No	27	−167
IG55	F	Yes	28	−185
IG44	F	No	29	−320
S13K	M	Yes	30	−337
IG7	F	No	31	−382
TK8	M	Yes	32	−451
S12K	F	Yes	33	−461
TK7	F	Yes	34	−493
S10K	F	No	35	−507
S9K	F	No	36	−561
S8K	F	Yes	37	−597
TK5	F	No	38	−638
S14K	F	No	39	−701
TK3	F	No	40	−714
TK6	F	No	41	−769
Blue11	F	Yes	42	−846
TK4	F	No	43	−851
S15K	F	No	44	−889
S16K	F	Yes	45	−964

Each day before we recorded the dominance interactions, the goats were allowed to feed for one hour in a rye grass pasture. This ensured that all goats had similar satiation levels and thus avoided situations where hungrier goats could be more aggressive. To determine the dominance hierarchy, we facilitated interactions between two goats at a feeding patch. For these interactions, a single artificial food patch (L×W×H: 57.0×36.5×23.0 cm) was provided. These patches contained 200 g of commercial sheep pellets (Complete Sheep Finisher, Meadow Feeds, South Africa) poured into one of the corners.

The main risk of injury that we foresaw during these interactions was an individual being cornered against a fence and thus being unable to escape from aggressive contact from the other goat. To prevent this, the interactions were conducted in a large (700×450 cm) pen, which allowed the subordinate goat to move away from the dominant individual after an interaction. Furthermore, we placed the feeding patch in the middle of the pen and released the goats simultaneously from opposite sides of the patch. This allowed the subordinate goat to decide whether it wanted to interact with the dominant goat, a situation that mimics natural conditions (Shrader, pers. obs.). If the subordinate goat did not approach the patch, we did not force an interaction but, rather, classified the feeding goat as dominant.

To initiate the interactions, we released the two goats simultaneously from ∼300 cm away on opposite sides of the patch. The goats were allowed to feed from the patch for up to 3 min. Dominance was determined during this time using visual cues [33], [34]. In no trial did the two goats interact aggressively for the entire 3 min period. Rather, a distinction between dominant and subordinate goats was generally made within the first 30 seconds.

Goats that consistently initiated aggressive acts and ultimately prevented or limited the feeding opportunities of the other individual were considered dominant. Dominant goats showed agonistic behaviour through aggression with contact, i.e. butting the flank or head of a feeding goat with their horns or the top of their heads if they did not have horns, and aggression without contact, i.e. threat displays and rush threats, see review by [38]. In contrast, some goats avoided a competitor completely and did not approach or feed from the artificial patch. We considered these individuals subordinate. With or without physical contact, it was easy to identify the dominant and subordinate goats via visual cues [33], [34]. Generally, if both goats approached the patch, vocalisations from the dominant goat were enough to cause the subordinate to retreat. Therefore, the agonistic interactions did not always result in physical encounters. In the limited instances (∼10% of the interactions) where aggressive physical interactions took place, these interactions were brief (<30 sec) and not severe enough to injure an individual. The maximum number of times that a dominant individual physically made contact with a subordinate before the subordinate retreated was three. Thus, extensive drawn-out aggressive confrontations did not take place during these interactions. In addition, to ensure that individuals were not hurt during the experiments, we observed all interactions between the goats from 500 cm away and thus could intervene and separate the goats if required. Moreover, there were full-time animal-handling staff on site that could assist and veterinarians on call in case of emergencies. However, no interventions or assistance was required due to the brief nature of the interactions.

Interactions were considered to be over once one of the goats withdrew [39]. Once dominance was determined, we removed the subordinate individual to reduce its stress and prevent any further unnecessary interactions. Dominant goats were allowed to continue feeding for the remaining portion of the 3 min to further familiarise them with the experimental setup. After the 3 min, we then released both the subordinate and dominant goats into a grass pasture to feed. This helped to ensure that these goats were not tested more than once a day. In all instances, when the dominant goats entered the pasture, they began feeding immediately and did not show aggravated levels of aggression towards the subordinate goat or the other herd members already within the pasture.

To generate the hierarchy, all individuals in the herd were tested against each other over a period of three months resulting in 1993 interactions. Dyads were chosen randomly because all goats ultimately interacted. Due to the size of the herd and the number of interactions, we only tested each dyad once. The majority of interactions resulted in a clear winner. However, if it was not clear which goat was dominant or subordinate, the two goats were retested on another day. If they still did not interact, we recorded them as being non-interacting [5]. Out of the 1993 interactions, only 13 were non-interacting, and these goats were not tested against each other in the patch-joining experiments (see below).

For each dyad, we identified the dominant and subordinate individuals and constructed a win-loss matrix to determine dominance [40]. The dominant individuals were assigned a value of one and subordinates zero. Dominance was calculated for each individual, using David’s score (DS) [41], [42]. We used DS because it deals logically with repeated interactions between group members and when there are equal numbers of interactions between dyads, it reduces row-sum scoring [43]. To determine the overall dominance status of each goat within the herd, the goats were ranked according to their DS value. We used the linearity index *h′* [44], and the win-loss matrix to measure the linearity of the hierarchy based on the dominance ranks from the DS values. When individuals in a dyad did not interact, each goat was given a value of 0.5 in our linearity calculations [44]. To avoid possible dominance-subordinate reversals [33], [45], we never tested consecutively ranked individuals against each other in the patch choice experiments.

The top six ranked goats within the herd were male (see Table 1) and patch-holders as well as patch-joining goats were a mixture of male and female goats. As a result, we included sex as a factor in the analysis to determine if it influenced patch-joining decisions. Two factors that were not included in the model were the presence/absence of horns and social bonds. Within the herd, 23 of the 45 goats had horns (51%). Because these individuals were found throughout the hierarchy, horns did not ensure that an individual was highly ranked (Table 1). In addition, the presence of horns did not influence how close individuals fed next to each other [46] and are therefore unlikely to influence patch-joining decisions. Moreover, because we were not testing for the factors that determined dominance, but rather were interested in the hierarchy itself, the presence/absence of horns was not included as a factor.

Due to the lack of information regarding the relatedness of the different goats in the herd, we could not consider social bonds in our analysis. However, due to this limited information, we may have potentially run tests between mothers and their offspring from previous years.

### Patch choice

To test for the effect of food availability in a patch, we used four artificial patch types, each with their own distinctive visual cues: black rectangle, white rectangle, black circle and white circle. Rectangular patches (L×W×H: 57.0×36.5×23.0 cm) were similar to the plastic trays used by [24]. We constructed smaller circular patches using a 45 cm diameter round plastic basin that was 10 cm deep. We used patches of different sizes to help the goats differentiate between the patches (i.e. the smaller containers represented smaller amounts of food). We used patch size as an indicator of food availability because herbivores can use sward height [47] and bush size [48] as a measure of food availability under natural conditions (e.g. large bushes likely contain more food than small bushes).

To provide diminishing returns within the different artificial patches and thus make them similar to natural patches [49]–[52], we attached an equally spaced 3×3 grid of 0.2 cm wire to the top of each patch. In addition, we added 5 ℓ of inedible medium, comprising of equal amounts of dried corncobs and a number of 20 cm lengths of 3 cm plastic tubing to the rectangular patches. The number of plastic tubes per tray was the same in each trial. In the smaller circular patches, we added 2.5 ℓ of the combined inedible medium. The difference in the amount of medium used between the different sized patches was relative to their respective volumes.

To generate patches of different food availability we put 400 g of commercial sheep pellets into the black rectangular patch, 200 g into the white rectangular patch, 100 g into the black circular patch and 40 g into the white circular patch. Finally, we created a ‘super patch’ by pouring 1000 g of food into a pile on a plastic sheet. This patch did not contain inedible medium because the amount of food completely covered the 5 ℓ of inedible medium used in the rectangular patch. Thus, it would not provide diminishing returns. Our choice of food quantity for the different patches was arbitrary. However, large differences in food availability were used between the patches to make it easier for the goats to identify and remember the different patch types.

During the eight months prior to experimentation, we tested whether the goats were able to visually differentiate between the patches (e.g. [53]). However, goats were not tested against the super patch. Therefore, we used the four different patch qualities, and tested each goat with the different patch combinations (black rectangle vs. white rectangle, white rectangle vs. black circle, etc.). The patch choice trials involved sequences of binary choices between two feeding patches made by individual goats across the different patch quantities. To ensure that the goats could differentiate among the patches, we tested all the goats within the herd twice for each of the six different patch combinations, resulting in 12 days of testing. Despite the majority of the goats being able to identify the best quality patches, we only used individuals as patch-joiners if they consistently differentiated between all the different patch quantity combinations. We did this to ensure that in our experiments, the patch-joiners were able to incorporate patch quality into their patch-joining decisions. The remainder of the goats then acted as patch-holders in the different experiments. However, we continued to test some of these goats throughout the experiment to see if any additional goats would be able to learn to differentiate between the patches. This was useful because some of the original goats needed to be replaced for the second experiment (see below).

**Figure 1:**
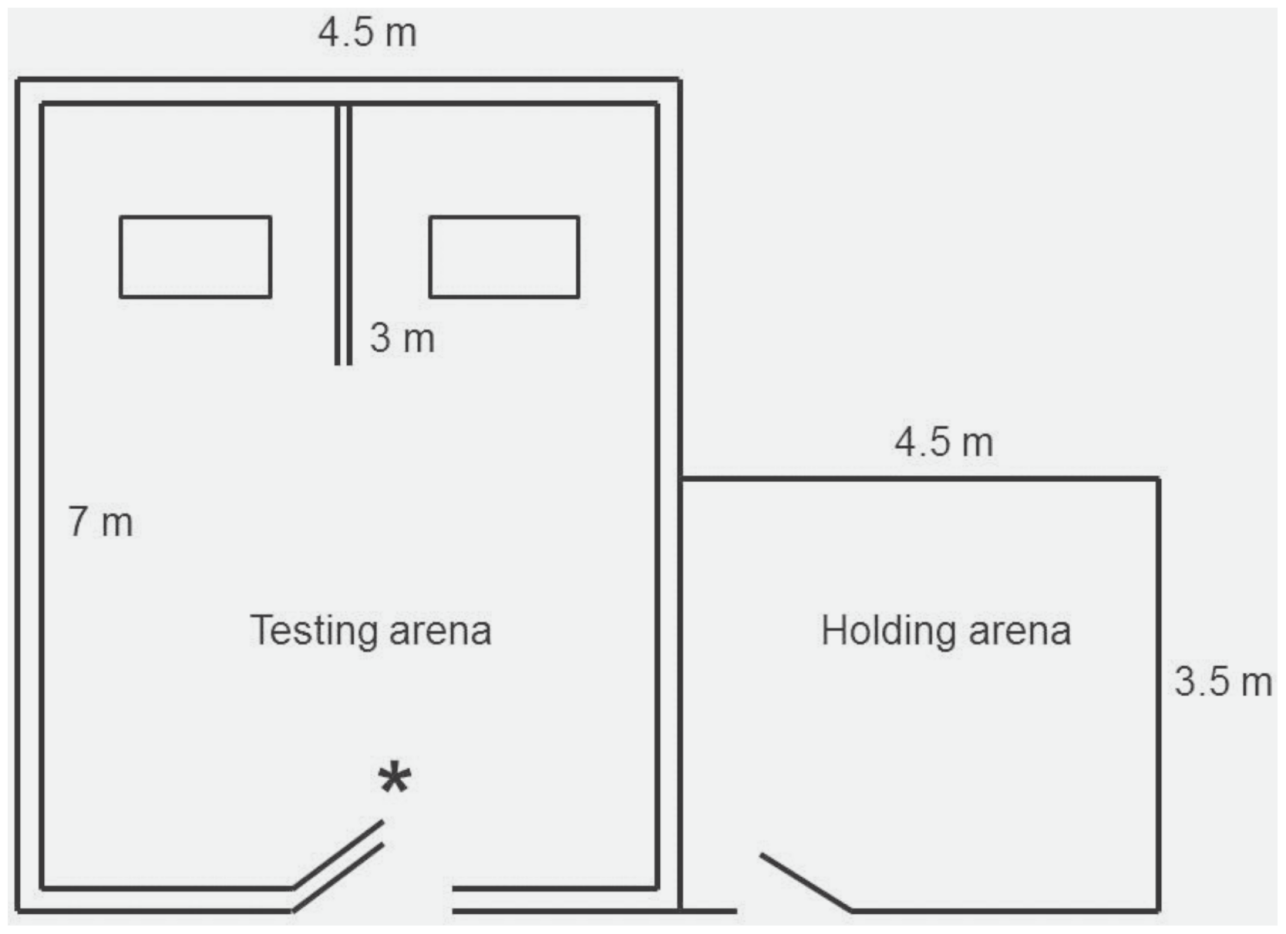
Testing arena used for the different experiments. Double lines denote non-transparent fences. Feeding patches (open rectangle) were placed on each side of the non-transparent divider. The * shows from where we released the patch-joining goat.

**Figure 2:**
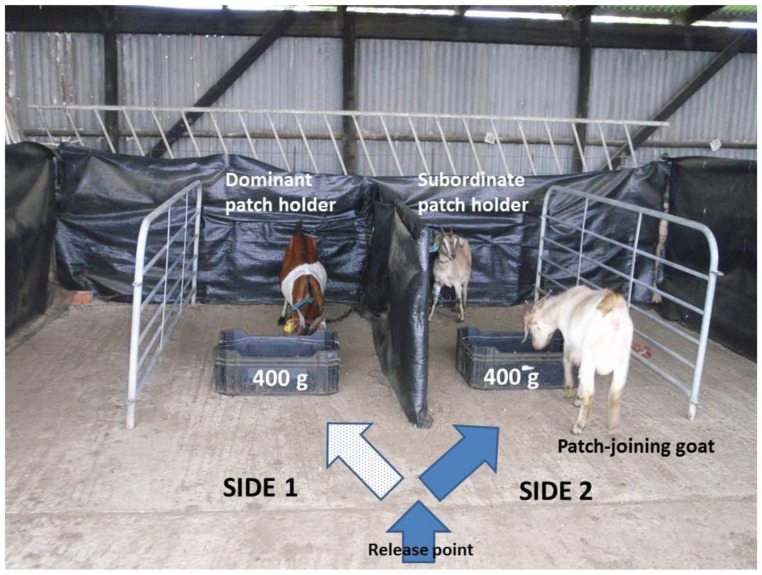
Annotated picture of the experimental design. Each patch-joining goat was released from the release point (∼4 m) from each patch. This gave the goats enough time to make a decision on which patch to join based off the dominant status of the patch-holders and the amount of food in the patch. Every goat went straight to a patch and started to feed for two minutes. During this time, no goat switched between trays. If the goat had followed the patterned white arrow, it would have joined the dominant patch-holder. Thus, in the above picture the goat decided to join the subordinate patch-holder.

We conducted these trials in a 700×450 cm pen with the outer walls covered with black plastic to ensure that the goats focused on the feeding patches (Figure 1). In addition, the end of the pen was divided in half using a non-transparent fence (300×150 cm) that ran from the back wall and placed one patch on either side of the divider. To ensure that the test goat had to enter a side in order to feed, we placed the artificial patches 30 cm inside of each half. This then allowed a subordinate patch-holder ∼150 cm of space to back away from an approaching dominant patch-joiner (see Figure 2) and, thus, limit aggressive interactions between these goats.

A patch-joining goat was released from the front of the pen from where it could see both patches. Once released, we recorded which side of the pen the goat entered. The criterion we used to determine whether a goat had entered a side of the pen was when it started to feed from the patch on that side. In each situation, the goat went straight to a side and started feeding. No goat investigated both sides before making a decision or switched between sides during the two minutes we allowed it to feed. To account for an area effect, the positions of the different patches were rotated between successive trials.

### Experiment 1: Patch food availability and the top ranked patch-holders

For the following experiment we used a subset of the goats (*N = *25, see results) as patch-joiners from the initial herd (*N* = 45) because these 25 individuals could successfully differentiate between all the different patch types. The remaining 20 goats, as well as some of the test goats, were used as patch-holders. To test the influence of food availability and dominance status of a patch-holder on patch-joining decisions of individuals, we simultaneously tethered a dominant and a subordinate goat behind separate artificial food patches, of the same quality, on either side of the divider (Figure 2). A subordinate patch-holder was any goat that was lower in rank than the patch-joining goat.

We initially only tested the top ranked goats in the herd because higher ranked goats continuously reinforced their status through aggressive interactions [33]. To determine which of the dominant goats were able to prevent patch-joining, we started with the highest ranked goat and moved down the hierarchy. Overall, we tested the top six ranked goats but found only the top four goats influenced the patch-joining decisions of individuals (see Results). Therefore, the top four dominant individuals (all males) were used in our experiments. In total, 25 different subordinate goats (male and female) were used in this experiment. As with the patch choice experiment, the test goat (i.e. the patch-joiner) was released from the front of the pen and we recorded the dominance status of the patch-holder it chose to join. As above, upon release each goat went straight to a patch and started feeding without hesitation. No individual investigated both sides before making a decision or switched between sides. Goats were allowed to feed for two minutes.

In contrast to the patch choice trials above, we did not provide the test goats with a choice between different combinations of patch types (e.g. 400 g vs. 100 g). Rather, we placed the same patch type in front of both the dominant and subordinate patch-holders in each trial (e.g. 400 g vs. 400 g). This allowed us to control for food availability and thus determine how dominance status of patch-holders influenced patch-joining decisions at each of the patch food availabilities. Another option could have been to use patches of different quantities in each trial, but this would have made it difficult to tease apart the factors driving patch selection. For example, if a goat joined a patch containing 400 g that was held by a subordinate, as opposed to a dominant individual’s 100 g patch, it would be unclear whether it was the amount of food or the subordinate status of the patch-holder that drove the patch-joining decision. Moreover, the use of different combinations of patches would require a greater number of trials than were run in our present experimental design. Thus, our experimental setup allowed us to minimise the number of tests that were required and, therefore, minimise any potential stress to the goats. As above, we only tested each goat once a day.

To limit discomfort of the patch-holders during this experiment, we tethered them to the back of the pen using a rope attached to a dog collar that we placed around their necks. The rope was long enough (200 cm) for the patch-holder to stand next to the patch, but did not allow it to feed. Although patch-holders were not able to feed, they still were, to some degree, able to defend their patch from goats joining them (e.g. rush and horn threats). Patch-holders were only tethered for two minutes per trial.

We tested each patch-joiner against each of the top four ranked goats at the different food quantities resulting in 20 days of testing. For each test, a different subordinate patch-holder was used (i.e. we never paired the same subordinate with the same top ranked goat across the different patch quantities). Furthermore, we switched the position of the dominant and subordinate goats between successive trials to control for a patch-joiner’s preference for a specific side of the pen. The position of the dominant and subordinate was randomly chosen for the first trial for each patch-joiner.

In addition, we explored whether the sex of the joining goat influenced patch-joining decisions. However, the small sample size of males (*N* = 4) in the herd of test goats prevented us from making any reliable inferences about the influence of sex. As a result, a more detailed analysis of the influence of sex on patch-joining decisions was conducted in the second experiment.

### Experiment 2: Patch quality and dominance status

To further explore the influence of dominance and patch quality, we ran an additional experiment. This allowed us to test the additional hypotheses that 1) dominance gap influences patch choice, 2) absolute rank of the dominant goat influences patch choice, and, finally, 3) the sex of the dominant and joining goat influences patch choice. We were able to test the influence of sex in this experiment due to the inclusion of three additional males (see below).

For this experiment, only 22 (7♂; 15♀) individuals were used that could differentiate between the different patches. These individuals comprised 19 (4♂; 15♀) of the goats used in the first experiment, and three additional males that, with supplementary training, had learned to differentiate between the patch types. We limited the addition of individuals to these three males, because during the initial training they were able to distinguish five out of the six patch quantity combinations. Moreover, the ranks of these males were from throughout the dominance hierarchy. Thus, their inclusion did not increase the number of either high or low ranking individuals. Ultimately, these males were added to increase the sample size because six of the goats from the previous experiment were removed. These removed goats preferred to jump out of the testing arena rather than feed from the patches in this experiment.

#### Hypothesis 1

To determine the influence of dominance gap and patch quality on patch choice, we used the same experimental design as in the previous experiment (i.e. simultaneously testing a patch-joiner against a dominant and subordinate patch-holder). Each patch-joining goat was tested three times against different dominant individuals that represented a range of different dominance gaps (i.e. close, intermediate and far in rank). The dominance gap was calculated between the patch-joiner and the dominant patch-holder. We considered this gap to be close if the dominant patch-holder was between 3–5 ranks above the patch-joiner, intermediate when these individuals were separated by 6–8 ranks and far if the separation was 9–12 ranks. Subordinate patch-holders comprised any goat that was lower in rank than the patch-joining goat. We subjected all the patch-joiners (*N* = 22) to each combination of three different dominance gaps at each of the five patch qualities, resulting in 15 days of testing. As above, we recorded which goat the patch-joiner chose to join (i.e. dominant or subordinate) at each of the different patch qualities. As with the previous experiment, goats were only tested once a day.

#### Hypothesis 2

To further explore dominance, we tested how absolute rank of the dominant patch-holder and patch quality influenced patch choice. The absolute rank is the rank of a goat in relation to the rank of all other goats in the herd. Goats were assigned an absolute rank in relation to their position within the dominance hierarchy (i.e. the top ranked goat had an absolute rank of 1 and the lowest ranked goat in the herd had an absolute rank of 45). We used the same experimental design as in the previous experiments. Each patch-joining goat (*N* = 22) was tested against three different dominant individuals that had different absolute ranks. Absolute dominance rank was restricted to a range of 1–36, as opposed to 1–45, because we only used 36 dominant individuals in the experiments. This was because 1) we needed to have goats that were subordinate to the lowest ranking dominant goat, and 2) we needed some goats to act as subordinate patch-holders.

#### Hypothesis 3

We assessed whether sex influenced patch-joining decisions because both male and female goats were used as dominant patch-holders as well as patch-joiners. Firstly, we separated the data into two categories: 1) when joining goats fed from dominant male patch-holders (*N* = 154), and 2) when joining goats fed from dominant female patch-holders (*N* = 176). We then asked whether the choice (i.e. to feed from a dominant or subordinate patch-holder) of male and female patch-joiners was influenced by the sex of the dominant patch-holder.

### Statistical analysis

Dominance calculations were run using DomiCalc [54], while all other statistical analyses were run with PASW (SPSS) v. 19 (IBM Inc.). We used *h′* to test the linearity of the dominance hierarchy [44]. As in other studies, the dominance hierarchy was considered linear when *h* ≥0.9 [40], [44]. The problem with arranging a win-loss matrix into a specific order is that it may create linear relationships where no such relationships exist. To determine the statistical significance of the linearity of the dominance hierarchy, a sampling process using 10,000 randomisations was used, see [44].

The patch choice experiment involved sequences of binary choices between two feeding patches. Because the same goats were used in all the experiments, individuals were treated as the subjects for repeated measures in generalized estimating equations (GEEs). We used GEEs because of the non-independence of the data [55]. Moreover, a GEE should be used if a subject is tested repeatedly and could potentially remember previous trials. Goats were tested over consecutive days, so not enough time elapsed between the trials for the autocorrelation of their behaviour to be reduced or absent. The model incorporated an exchangeable correlation matrix, binomial error distribution and a logit link function and significance was analysed using score statistics. For graphical representation, we back-transformed data from the logit scale which resulted in asymmetrical confidence intervals (CIs). To assess if goats could visually differentiate between the patches differing in food availability (i.e. food quantity), we analysed the proportion of visits made by goats to the higher food quantity patch offered in the choice experiment. We used means and their 95% CIs to determine whether goat’s preference between patches departed from the expected 50% visit to each patch type under random visitation for each food quantity combination.

We used GEEs to determine whether the rank of the four most dominant goats influenced patch-joining decisions. GEEs were used because choices were non-independent because all the 25 joining goats were tested against the same top ranked goats at each patch food quantity. These were choice experiments, so we compared the number of patch-joining goats that avoided the dominant patch-holder at each food quantity. The response variable was choice (i.e. 1 =  goat fed from a dominant patch-holder, 0 =  goat fed from a subordinate patch-holder) and the factors that were included were rank of the dominant patch-holder (1–4), food availability (40 g, 100 g, 200 g, 400 g, 1000 g) and their interaction. As the amount of food increased, fewer of the top ranked patch-holders were avoided (see Results). By fitting a logarithmic trendline to these data (y = −1.1611Ln(x) +8.3519), where y is the top ranked goat that is avoided and x is the amount of food, we were able to predict when the quantity of food became more important than patch-holder dominance. Moreover, we used this trendline to generate behavioural titrations to predict how much better a patch needed to be for a patch-joiner to join a higher ranked patch-holder.

To explore the influence of dominance gap, absolute rank and patch quality on patch-joining decisions, we ran two GEEs. We used GEE, because it is a conservative test that takes into account the possible non-independence of the data [55]. For the dominance gap model the response variable was choice (i.e. 1 =  goat fed from a dominant patch-holder, 0 =  goat fed from a subordinate patch-holder) and the factors were patch food availability (40 g, 100 g, 200 g, 400 g, 1000 g), dominance gap (between 2 and 20 individuals) and their interaction. The absolute rank model had the same response variable but the factors were patch food availability (40 g, 100 g, 200 g, 400 g, 1000 g), absolute rank and their interaction.

To determine whether food availability, the sex of the joining goat and their interaction influenced patch-joining decisions we ran a separate GEE for male and female dominant patch-holders. The sex of the joining goat did not influence how they reacted to a dominant male or female patch-holder (see Results). As a result, we pooled the data and ran a GEE to determine whether the sex of the dominant patch-holder influenced the choice of the joining goat across the different patches. This allowed us to determine whether there was a difference in the proportion of goats joining a dominant or subordinate patch-holder when the dominant goat was male or female.

## Results

**Figure 3:**
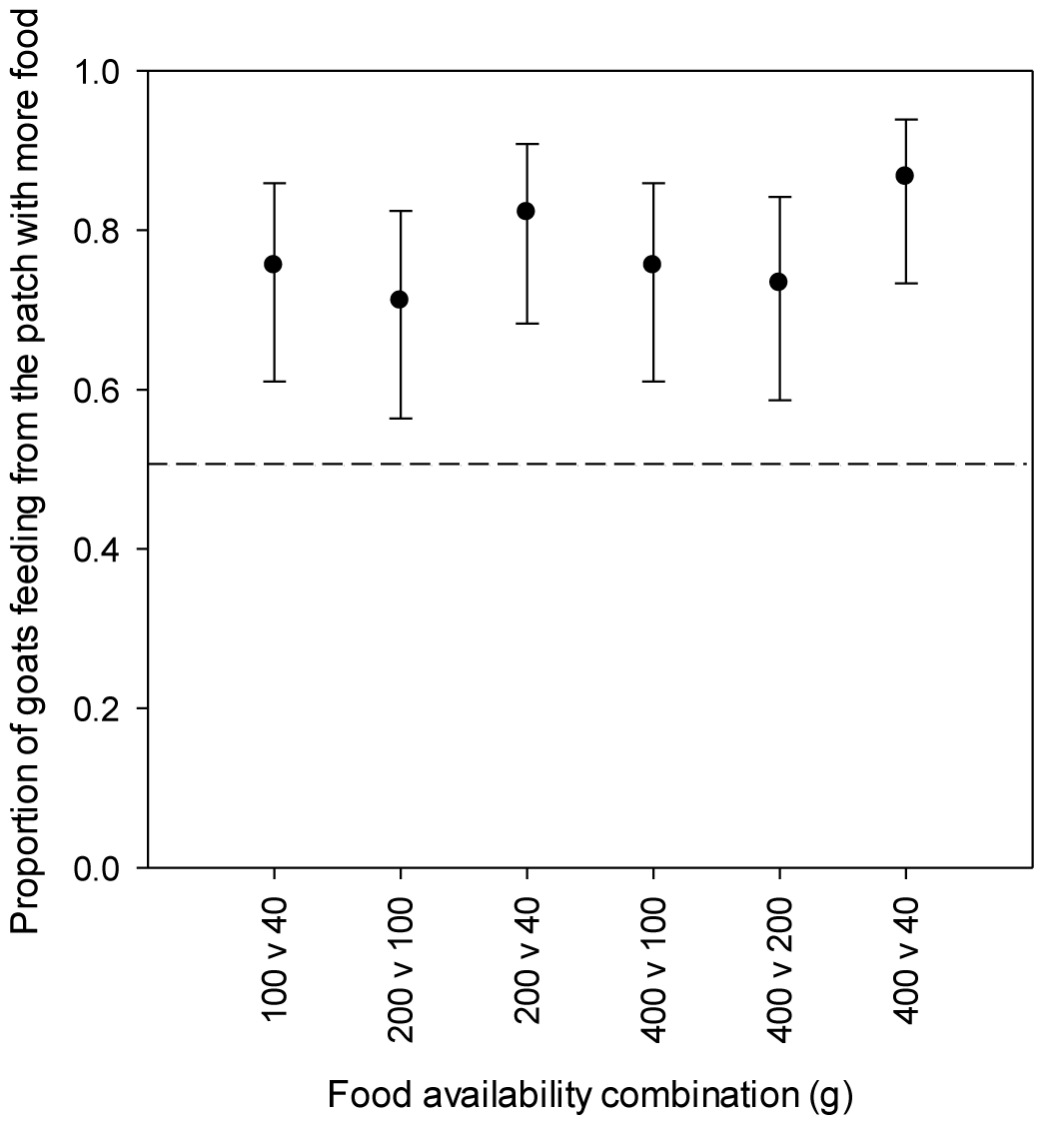
Patch-joining goats prefer to feed from the patch containing more food at each food availability combination. Marginal means (±95% CI) of the proportion of visits to the patch with the highest food availability are plotted. At each food availability combination, there is no overlap with the 0.5 expectation under random visitation, indicating a preference for the patch with the most food.

The interactions from the win-loss matrix confirmed a linear dominance hierarchy (*h′* = 0.96) that was statistically significant (Linearity test using *h′*, based on 10,000 randomisations, *P*<0.001). In the patch choice experiment, goats showed a clear preference to feed from the patch with the largest food quantity in each food availability combination (Figure 3). The better patch (i.e. more food) received between 71% and 87% of all visits, which differed significantly from the expected 50% under random visitation (Figure 3). From the herd (*N* = 45), 25 individuals (4♂; 21♀) consistently selected the patch with more food across all patch combinations. We used these individuals as patch-joiners in the patch choice experiment against the top ranked goats.

### Experiment 1: Patch-joining against top ranked goats

**Figure 4:**
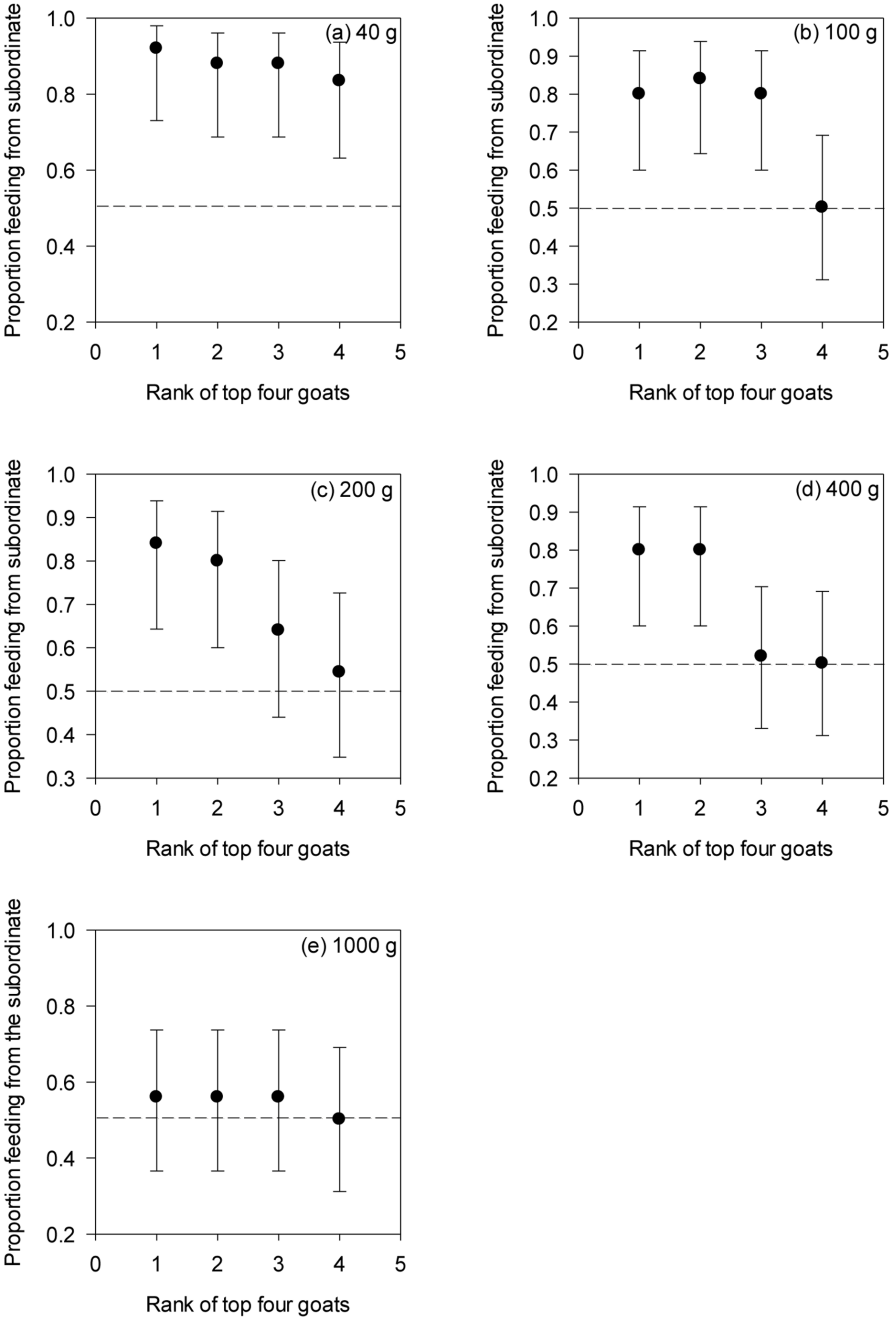
Proportion of goats feeding from a subordinate when tested against the top four ranked goats. Back transformed marginal means (±95% CI) of the proportion of goats feeding from a subordinate patch-holder when tested against each of the four most dominant patch-holders. Patches comprise (a) 40 g, (b) 100 g, (c) 200 g, (d) 400 g and (e) 1000 g. No overlap at each rank between the 95% CI and the 0.5 expectation under random visitation indicates an avoidance of that dominant patch-holder. Overlap between the 95% CI and the 0.5 expectation indicates a lack of preference with patch-joining goats joining both dominant and subordinate patch-holders.

**Figure 5:**
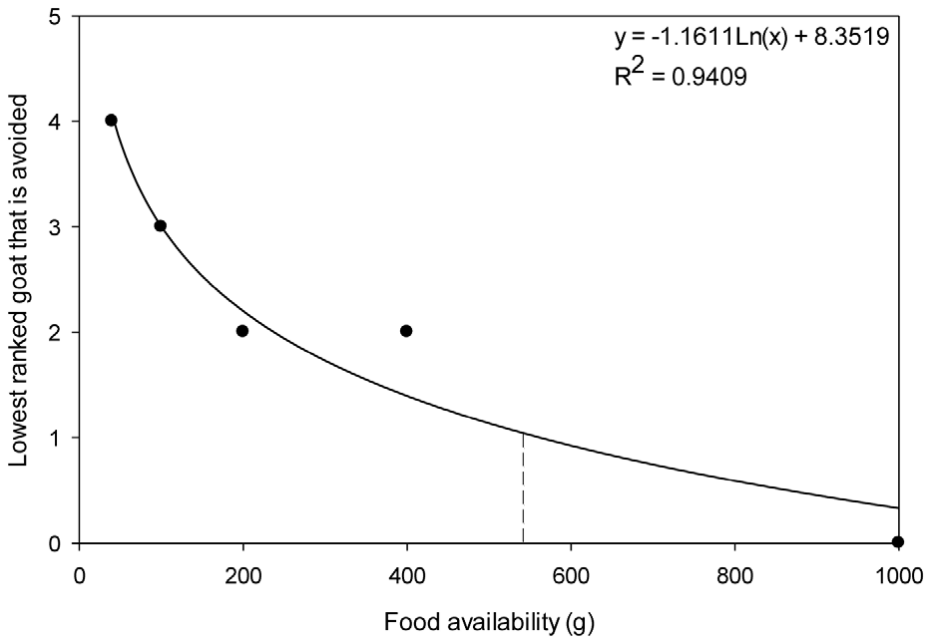
Change in the number of top ranked individuals avoided by patch-joiners as food availability increases. Y-axis represents the lowest ranked goat of the top four ranked individuals that was avoided. Ranking is set so that rank 4 indicates that the top four highest ranked goats were avoided, 3 the top three, 2 the top two and 1 only the top goat was avoided. Dashed line indicates the amount of food (∼563 g) where the most dominant individual was not avoided by patch-joining goats, and thus where the quantity of food within a patch overcame dominance as the main factor influencing patch-joining decisions.

When we tested patch choice of goats against the top four ranked individuals, we found significant effects of rank (GEE: χ^2^ = 28.118, *P*<0.001), food availability (GEE: χ^2^ = 15.419, *P* = 0.004) and their interaction (GEE: χ^2^ = 21.019, *P = *0.05). Interestingly, individuals avoided high-ranking goats as a function of food availability (Figure 4 a–e). When food availability was low, goats avoided all dominant individuals. However, as the amount of food in the patches increased, the number of dominant individuals that were avoided decreased. This continued up to the highest patch quantity (i.e. 1000 g), where dominance rank did not influence patch-joining decisions, and individuals equally joined top ranked individuals and subordinates at their patches (Figure 5). By fitting a logarithmic trendline to these data (y = −1.1611Ln(x) +8.3519), where y is the top ranked goat that is avoided and x is food availability, we were able to predict that at ∼563 g, the amount of food in a patch becomes more important than patch-holder dominance status with regard to the patch-joining decisions of our goats (Figure 5).

We generated titrations for our study by first determining the patch qualities at which individuals would join the different top ranked goats by using the trendline from our results (y = −1.1611Ln(x) +8.3519). This resulted in estimates of 563 g for the top ranked patch-holder, 238 g for the second highest, 100 g for the third and 42 g for the fourth. Then by dividing the estimated amount of food in a patch required to join a more dominant patch-holder (e.g. 563 g) by that required to join the next lower ranked patch-holder (e.g. 238 g), we found that a patch needed to be ∼2.4 times richer for individuals to consider these two patches to be of equal value.

### Experiment 2

#### Hypothesis 1 and 2: Influence of dominance gap and absolute rank on patch-joining

Contrary to Experiment 1, when we expanded our investigation to include patch-joining decisions across a range of dominance gaps, goats joined patches held by both dominant and subordinate patch-holders equally. As a result, there was no effect of food availability in a patch (GEE: χ^2^ = 7.366, *P* = 0.118), dominance gap (GEE: χ^2^ = 0.759, *P* = 0.385) or their interaction (GEE: χ^2^ = 7.390, *P* = 0.117) on patch-joining decisions. Furthermore, patch-joining decisions were not influenced by the food availability (GEE: χ^2^ = 7.575, *P* = 0.108), absolute rank of the dominant patch-holder (GEE: χ^2^ = 0.389, *P* = 0.533), or their interaction (GEE: χ^2^ = 8.657, *P* = 0.070).

#### Hypothesis 3: Sex and patch choice

The sex of the joining goat as well as the sex of the dominant goat did not influence patch-joining decisions. When joining goats only joined dominant male patch-holders, there was no significant effect of food availability (GEE: χ^2^ = 6.214; *P* = 0.184), sex of the joining goat (GEE: χ^2^ = 1.888; *P* = 0.184) or their interaction (GEE: χ^2^ = 7.734; *P* = 0.102) on patch-joining decisions (i.e. male and female goats joined both dominant and subordinate patch-holders equally). Moreover, the same result was found when the dominant patch-holder was female (Food availability: GEE: χ^2^ = 1.742; *P* = 0.783, Sex of joining goat: GEE: χ^2^ = 0.739; *P* = 0.390, Food availability X sex of joining goat: GEE: χ^2^ = 7.701; *P* = 0.103). Because male and female patch-joiners did not react differently to male and female dominant patch-holders, we pooled the above data. This then allowed us to determine whether the sex of the dominant patch-holder influenced the proportion of patch-joining goats that joined a dominant or subordinate goat. Once again, food availability (GEE: χ^2^ = 4.692; *P* = 0.320), the sex of the dominant goat (GEE: χ^2^ = 0.520; *P* = 0.471) and their interaction (GEE: χ^2^ = 6.074; *P* = 0.194) did not influence patch-joining decisions.

## Discussion

The patch-joining decisions of herbivores can be influenced by both the quantity of food in a patch and the dominance status of patch-holders [12]. However, until now, the combined effect of these two factors has been poorly explored. Generally, individuals prefer to join patches held by subordinate individuals [14], [56], [57]. In contrast, results from Experiment 1 suggest that goats only joined subordinate patch-holders when the dominant patch-holders were the top ranked goats in the herd. However, as food availability in the patches increased, goats avoided fewer of these top ranked individuals. This suggests that when facing the most dominant goats when the amount of food in a patch is low, patch-holder dominance status dictates patch-joining decisions (i.e. costs of aggression are greater than the potential benefits of food intake). However, as food availability increases, eventually the benefits of feeding in the patch outweigh the costs of aggressive interactions with even the most dominant of patch-holders.

When we tested patch-joiners’ responses to the patch-holder being subordinate or the four most dominant goats, we found that as predicted in social foraging models [14], [56], goats preferred to join patches held by subordinate individuals. However, when the dominant patch-holder was from elsewhere within the hierarchy, individuals seemed to ignore patch-holder rank (i.e. dominance gap and absolute rank) and equally joined both subordinate and dominant patch-holders (Experiment 2). As a result, this greatly increased the number of patches that individuals could join, and thus increased feeding opportunities. Therefore, it seems that for the goats only the top ranked individuals (in our case the top 10%, *N = *4) were dominant enough to prevent individuals from joining their patches (Experiment 1). However, this ability declined as the amount of food within the patch increased.

One explanation for increasing food availability overriding patch-holder rank could be that at low food availability, the finder’s share comprises a large proportion of the available food [20]. This then leaves very little food for the patch-joining goat when it joins the patch. Furthermore, in low food quantity patches, patch-holders are likely to defend the small amount of food within the patch more aggressively [21]. As a result, within these patches, the costs of joining a dominant patch-holder greatly outweigh the potential benefits [11]. However, as the amount food in the patches increases, the amount of food available to a patch-joining goat (i.e. benefit) increases and the intensity of patch defence (i.e. cost) declines.

Behavioural titrations provide a method to quantify dominance with regard to patch use behaviour [25]. In general, individuals should join a patch when the marginal benefits (e.g. the amount of food) exceed the marginal costs (e.g. rank of the patch-holder increases) [25]. We found that the initial amount of food needed to be ∼2.4 times richer for individuals to consider these two patches to be of equal value. Thus, at that point, the marginal benefit of joining either patch was the same.

In Experiment 2, when we explored the rest of the dominance hierarchy, neither dominance gap nor absolute rank of the dominant patch-holder influenced patch choice. This was surprising because individuals generally tend to avoid feeding from a dominant individual’s patch [12], [14], [34], [58]. This could be due to the patch-holders being tethered and thus not posing a threat to the patch joiners. However, we observed patch-holders being aggressive towards patch-joiners (e.g. horn threats and vocalisations) that attempted to approach the feeding tray. Thus, this suggests that the threat of aggression was real.

Another, potentially more likely explanation for patch joiners not avoiding dominant patch-holders could be that the dominance gaps we used were not large enough for dominance to be important. In our experiments, the size of the dominance gaps was limited by herd size as well as by the number of goats that could differentiate between the different patch qualities. Therefore, dominant patch-holders did not necessarily include the top ranked individuals of the herd. Thus, many of the goats would have faced dominant patch-holders that were intermediate in rank overall, and thus not pose enough of a threat to prevent individuals from joining a patch. For example, Barrosso *et al.* [33] found that intermediate ranked goats were less aggressive than top ranked individuals. This may explain why the goats in our study joined both dominant and subordinate patch-holders when the dominant goat was from the middle of the hierarchy. Moreover, it may also explain why the absolute rank of the goats did not influence patch-joining decisions.

Interestingly, our results contradict the findings of a number of studies that suggest that the absolute difference in dominance between individuals is important in patch choice [13], [58]. For example, Mexican jays (*Aphelocoma ultramarine*) tend to join patches more frequently if they are held by lower ranking individuals compared to patch-holders that are closer in rank [12]. In addition, other studies have shown that patch-joining individuals only join subordinates at their patch [14], [56], [57]. However, the majority of this research has focused primarily on birds [57]. Our contrasting results may be due to us focusing on a mammalian herbivore. This suggests that it is difficult to make generalizations about the role of dominance in determining patch choice across taxa, see [59].

For herbivores, food patches (e.g. grass or a shrub) are not very discrete and are thus difficult to defend [40]. When dominant individuals are not able to defend patches, they cannot prevent other individuals from joining the patch [59]. Our results suggest that the only individuals capable of defending a patch were the most dominant individuals. As a result, within a herd there are likely numerous patch-joining opportunities for individuals irrespective of their rank.

The fact that only 25 goats were able to differentiate between all six patch quantity combinations, suggests that they may have had difficulty differentiating between such wide ranges of food quantity combinations. Alternatively, there may have been too many factors (i.e. colour, size and shape) associated with the different food availabilities. If fewer of these variables were used, it is possible that more of the goats could have consistently selected the patch with more food.

Finally, due to the limited information on social bonds, related goats may have been tested against each other. The relatedness between goats can influence foraging decisions, with related individuals feeding closer to each other and thus not interacting aggressively [46]. However, in our dominance relationship experiments, all goats interacted. The 13 non-interacting pairs were never tested against each other in the patch-joining experiments. Therefore, if we tested related goats, then they too displayed dominance behaviours and limited the feeding opportunities of the related subordinates. Thus, if we tested related individuals, it unlikely influenced our results.

## Conclusions

An additional outcome of our study is that our results create new opportunities to expand aspects of social foraging theory [7] (e.g. producer-scrounger models). Specifically, as we have focused on a group-living mammalian herbivore, our findings better incorporates this important group into this theory. Moreover, our findings highlight the importance of food availability for these herbivores, and potentially other species, when they make patch-joining decisions. Furthermore, although we did not directly test producer-scrounger interactions, because the goats did not produce, the fact that subordinate individuals joined dominant patch-holders challenges the assumption of these models that individuals can only scrounge from subordinates. By expanding scrounging opportunities for individuals within the models to include scrounging from dominant individuals (except possibly the top 10% of the hierarchy) and incorporating food availability, social foraging theory could be expanded to better explore the foraging decisions made by group-living mammalian herbivores. In addition, our findings on herbivores may help explain why patch-holder dominance sometimes fails to explain patch-joining decisions of other species in producer-scrounger models [28], [60], and suggest that certain predictions of the models may not apply to all taxa.

## Supporting Information

Data Supplement S1 (XLSX)
